# A New Approach to Medical Records

**Published:** 1990-03

**Authors:** Denis Pereira Gray, Michael S Hall

**Affiliations:** Department of General Practice, Postgraduate Medical School, University of Exeter; Department of General Practice, Postgraduate Medical School, University of Exeter


### A New Approach to Medical Records

**Professor Denis Pereira Gray and Dr Michael S Hall**
Department of General Practice, Postgraduate Medical School, University of Exeter

### THE CARE CARD PROJECT

For many years the use of high technology information in banking has been widespread. Millions of people now hold credit cards and they have become a common and familiar part of the scene whenever financial transactions take place.

Banking is about to move on to new technology, involving instant debiting of the customer's account electronically and a new set of cards for which annual fees will have to be paid.

Health service technology has lagged somewhat behind, partly because of the much lesser investment and partly because the same energy drive has not been devoted to medical records as to banking records.

It is therefore a pleasure to report that an exciting and radical research project has been undertaken in the West of England, in the Department of General Practice in the Postgraduate Medical School of the University of Exeter. The so-called Smart Card Project, now called Care Card, represents the introduction to medical record keeping of a card similar to a credit card. The card contains a 16 kilo bit electronic chip which, when linked through a card reader, is a computer. Using a coding system, data are stored in compacted form and contain the patient's medical record, which can be read at a variety of sites in the National Health Service. Essentially the card will be organised through the patient's own general practitioner, who will be responsible for producing the basic set of data. More and more general practitioners are already holding such information in computer form. Nearly half of all general practices in the West of England now have computers.

Giving the patient his medical record breaks through a number of traditional barriers and helps to emphasise the importance of the patient.

### ADVANTAGES

First of all it empowers patients by giving them access to their own medical record: they can see it, read it, and could choose to obtain the readers which give them access to it.

The Care Card Project will underline the enormous importance of the population base of the registered list of patients with general practitioners. Despite numerous problems in accuracy over changes of address and other details, this is still far and away the most accurate and important population base in the British National Health Service. It is, for example, already used for the cervical cytology programme and is going to be used for the breast care cancer screening programme.

Indeed, this development is likely to improve dramatically the accuracy of medical records because there is nobody who has a greater interest in their accuracy than the patients themselves. Ninety-eight per cent of the British population are registered with general practitioners under the National Health Service and patients are more likely to inform their own family doctor of a change of address (if only because they may want him to visit them at home!) than any other agency. Basing a record system on the general practitioner's records is therefore likely to produce the best available system.

Another gain will be that it will provide important secondary advantages for hospital services. For example, it will enable rapid access to information in emergencies. If a patient is carried unconscious into hospital the hospital doctor in the accident and emergency department will be able to access immediately the crucial information he needs about allergies, medications and diseases known at present only to general practitioners. This may provide a whole variety of gains in terms of previously known adverse reactions to drugs, but allergies, current treatments and main medical diagnoses are likely to be the principal factors revealed.

Finally, the introduction of a care card held by the patient but used both in general practitioner premises and hospital premises is likely to help reorientate doctors and allied health professionals towards the basic concept of partnership between clinicians. The worlds of general practice and hospital medicine are still too separate, but if sharing manual co-operation cards has already helped, surely an electronic card will do more to build up a sense of partnership between doctors and nurses in all parts of the National Health Service?

### PROBLEMS

Despite all these considerable advantages there are of course problems to be examined and surmounted. The cards have a significant cost, in large scale production perhaps about £1 each, staff will need to master keyboard handling to enter information and to read it, and there will be problems of ensuring that information is up-to-date. Patients may lose their cards and there may be difficult issues to resolve about confidential information, particularly in relation to psychosocial problems.

### NEED FOR EVALUATION

These are the reasons why it is important that such an important advance, which has been pioneered in England by Bull Information systems and supported by the Department of Health, should be rigorously evaluated by an academic department able to weigh the pros and cons and to make suggestions about future developments.

The Department of General Practice in Exeter is extremely pleased that following its successful evaluation of the Micros for GPs Scheme for the Department of Health, it has now been given this important contract and it looks forward with great enthusiasm to evaluating it in the field tests.

### LAUNCH BY MINISTER OF HEALTH

On 1 March 1989 the Minister of Health, Mr David Mellor, came to Exeter to open the pilot study. He registered himself as a National Health Service patient with Dr Robin Hopkins, General Practitioner, Exeter and Research Fellow in the Department of General Practice and went on to a pharmacy to receive a Health Service prescription written for him by the doctor – and the whole system worked admirably. Mr Mellor was joined at lunch by a large number of leaders in the National Health Service and university world and then went on to the Department of General Practice where he met a group of general practitioners.

### CONCLUSION

The Smart Card/Care Card technology represents an interesting example of the impact of the third industrial revolution which is now evolving and infiltrating almost every aspect of professional practice. It is a good example of how high technology advance can penetrate and percolate into all aspects of medical care and it is now possible to envisage all patients in the National Health Service holding their own medical records in this form within the foreseeable future.

